# Corpus Callosum Volume in Treatment-Resistant compared to Treatment Responsive Schizophrenia patients with and without cannabis use disorder: a Novel Artificial Intelligence Method Applied to Single-Subject Magnetic Resonance Imaging

**DOI:** 10.1192/j.eurpsy.2025.768

**Published:** 2025-08-26

**Authors:** M. Matrone, A. Romano, G. Kotzalidis, F. Perrini, M. Ciccarelli, L. Vellucci, A. Barone, F. Iasevoli, S. De Persis, A. de Bartolomeis, A. Bozzao

**Affiliations:** 1 Neurosciences, Mental Health, and Sensory Organs Department (NESMOS), Sapienza University of Rome, Faculty of Medicine and Psychology, Rome; 2 Department of Mental Health Protection and Promotion, Unit of Addiction Pathology, Rieti; 3 UOC DSMDP District, TSMREE ASL ROMA 6, Velletri; 4 Diagnostic, Research and Training Centre in Cognitive-Behavioral Psychotherapy, A.T. Beck Institute, Rome; 5 Unit for Treatment Resistant Psychoses, Section of Psychiatry, Department of Neuroscience; 6 Department of Translational Medical Sciences; 7 Staff of UNESCO Chair on Health Education and Sustainable Development, University of Naples “Federico II”, Naples, Italy

## Abstract

**Introduction:**

The corpus callosum (CC) is essential for interhemispheric communication, and its abnormal integration is central to the neurobiology of schizophrenia (SCZ). SCZ patients have a 10-fold higher risk of cannabis use disorder (CUD) and about 20-35% show a lack or poor response to antipsychotics and are defined as treatment-resistant schizophrenia (TRS) Until now, no study has analyzed the morphology of the CC in TRS compared to healthy controls (HC) and non-TRS patients with and without CUD.

**Objectives:**

The aim of the study is to assess whether the diagnosis of psychosis, the response to antipsychotic treatment, and CUD can influence the volume of the CC. To achieve this, we used an innovative artificial intelligence program applied to MRI, which provides structural information on a single subject.

**Methods:**

We included 20 HC and 48 SCZ patients, of whom 14 were affected by TRS and 34 were non-TRS. Among the non-TRS group, 20 had CUD comorbidity (non-TRS-CUD^+^) and 14 did not have CUD (non-TRS-CUD^-^). All were assessed cross-sectionally through the Neurological Evaluation Scale, the Brief Assessment of Cognition in Schizophrenia, the Positive And Negative Syndrome Scale. We assessed them cross-sectionally using psychometric tools, cognitive tests. All patients underwent a brain MRI 1.5 T, for white matter volume group analysis, and MRI applied to Artificial Intelligence (MRI-AI-Pixyl.Neuro) for single-subjects analysis.

**Results:**

TRS was associated with higher PANSS total score (fig. 1) and neurological soft signs (fig. 2) and lower negative symptoms (trend) than non-TRS groups. The TRS group performs worse in the Tower of London task compared to non-TRS and HC groups. Only the condition of TRS is associated with a significantly smaller CC volume (64.28%) compared to HCs and non-TRS patients (Fig. 3). Only one patient from the non-TRS-CUD^-^ group showed a reduction in the volume of the CC like TRS patients.

**Image 1:**

**Figure:**
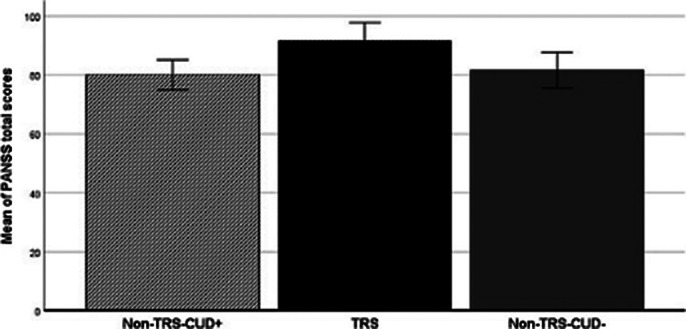

**Image 2:**

**Figure:**
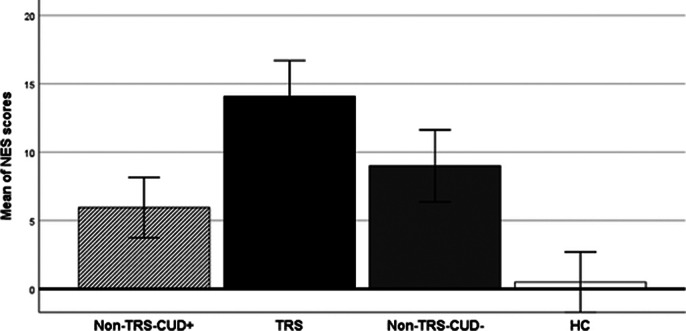

**Image 3:**

**Figure:**
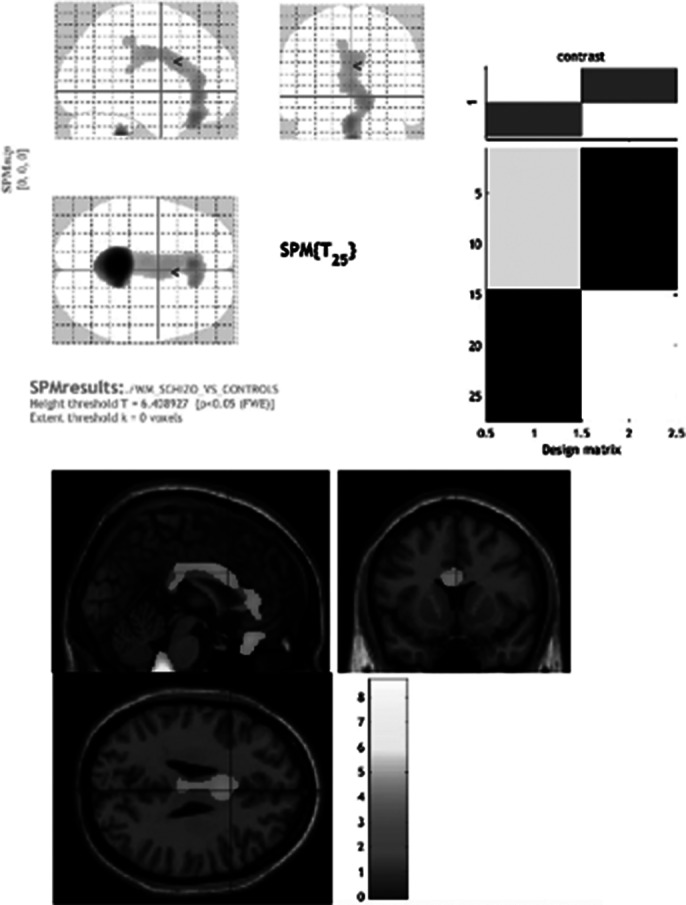

**Conclusions:**

TRS is associated with more severe general and negative symptoms, NSS, and cognitive dysfunctions and with a significantly smaller CC volume, demonstrating the role of this structure in the pathogenesis of TRS and probably in executive function impairment. Is conceivable that TRS has unique evolution and course characteristics, and that continuous cannabis use for 6.95 years is probably not sufficient to cause the structural alterations typical of TRS.

The MRI-AI applied to a single subject has shown reliable results, confirmed by classical group analysis, and represents a revolutionary tool for identifying potential neuroradiological biomarkers of disease, enabling quick TRS diagnosis in clinical practice, faster clozapine treatment following TRIPP guidelines, and easy application using only a standard volumetric sequence without post-scan analysis.

**Disclosure of Interest:**

None Declared

